# Supplementary material for: Are carbon emissions trading and green financial instruments synergistic? -Comprehensive quantitative research based on content analysis
Source: PLoS One. 2024 Mar 7;19(3):e0298601. doi: 10.1371/journal.pone.0298601 (PMC10919601; doi:10.1371/journal.pone.0298601)
Supplement: S1 Data — (ZIP) [file pone.0298601.s001.zip › Data/地级市Z_F工作报告.zip!/╔╜╢1⁄2/╠⌐░▓╩╨/└┤╫╘╣1⁄2╓┌║┼í╛┬φ┐╦╩2╛▌═°í┐_2005.docx]

各位代表：

现在，我代表泰安市人民政府，向大会作政府工作报告，请予审议，并请市政协各位委员和其他列席会议的同志提出意见。

一、过去的一年，全市经济与社会发展取得显著成绩

过去的一年，在市委的坚强领导下，全市人民高举邓小平理论和“三个代表”重要思想伟大旗帜，认真贯彻党的十六届三中、四中全会精神，紧紧围绕建设经济强市目标，以科学发展观总揽全局，坚持“四个坚定不移”，继续突出“三大重点”，切实抓好“五个着力点”，振奋精神，团结拼搏，埋头苦干，强力推进各项工作，全市经济与社会保持了持续快速健康发展的良好势头。

经济发展速度效益同步快速提高。初步预计，全市实现生产总值732.1亿元，比上年增长16.4%，其中一二三产业增加值分别增长7.1%、20.9%和13.7%。完成地方财政收入31.2亿元，同口径增长26.3%，尤其是财政收入结构发生了明显变化，地方税收收入增长40.9%，占地方财政收入的比重同口径提高6.67个百分点。规模以上工业实现销售收入690亿元、利税75.7亿元、利润36.7亿元，增长47%、68.9%和80.7%。民营经济实现增加值312.4亿元，增长35.2%；上缴税金22.5亿元，增长42.1%。实现社会消费品零售总额224.2亿元，增长15.5%；实现国内旅游总收入46.7亿元、旅游创汇2910万美元，增长37.4%和100%；进出口总额达到6亿美元，其中出口4.5亿美元，增长41.9%和45.1%。

解决“三农”问题成效显著。农业特产税免征、农业税税率降低3个百分点，全市农民人均减负68.1元；3905万元粮食直补和400万元的良种补贴，及时足额兑现到农户。全年粮食总产增长3.8%，新发展无公害农产品、绿色食品、有机食品认证面积21.4万亩、16.2万亩、2.1万亩，尤其是“奶业富民”工程大力推进，奶牛存栏量6万头，增长68%。引进、新建规模较大的农业龙头企业30多家，其中投资过亿元的8家。新增市外劳务输出9.1万人，累计达到45.8万人。“村村通油路”新增里程2108.4公里。新型农村合作医疗试点受益农民达30余万人，防治禽流感、防汛和排涝救灾工作取得显著成果，农田基本建设、植树造林、农业开发、农机等工作都取得新的成绩。

项目建设取得明显成效。新开工投资5000万元以上的工业、商贸、旅游项目127个，投资规模171.8亿元。其中投资过亿元的项目61个。泰山玻纤公司2万吨无碱玻璃纤维生产线、鲁能泰山电缆、钴酸锂电池材料、中科2万吨碳酸二甲酯、石横特钢60万吨高速线材、300万吨大水泥、知心仁食品、弘德机械等51个产品销售收入过亿元的工业项目建成投产，形成新的税源，成为调整产业结构、拉动经济增长的重要力量。

社会事业全面协调发展。全年获省以上科技奖励59项，国家重点和省级高新技术企业发展到17家和152家，高新技术产业产值占规模以上工业总产值的比重达到19.8%，提高3.4百分点。以县为主的农村义务教育管理体制初步确立，“两基”成果得到巩固提高，各类教育发展加快，办学质量和效益有新的提高，高考重点本科上线人数居全省第三位。驻泰高校、科研机构在服务地方经济社会发展中取得显著成绩。爱国卫生运动深入开展，公共卫生“两个体系”建设得到加强，市和各县市区传染病医院“新改扩”建工程基本竣工。人口和计划生育工作在巩固中提高，人口自然增长率为7‰。群众性精神文明创建活动深入开展，实施“四五”普法教育和“三五”依法治市取得积极成效。厂务、村务、政务公开和社区建设、村民自治得到加强，双拥共建取得新成绩，我市又一次被评为全国双拥共建模范城市。文化、体育、广播电视、新闻出版、外事侨务、民族宗教、人防、气象、史志、档案、老龄、妇女、儿童和残疾人等事业都有新进展。

群众切身利益得到较好保障。做好就业再就业、健全社会保障体系、实施少数民族帮扶致富工程等9件实事取得显著成效。全市新增就业再就业8.1万人，城镇登记失业率为3.6%；以养老、失业、医疗等保险为主要内容的城镇社会保障体系基本建成，各项社会保险费收缴率达到95%以上，企业离退休人员养老金社会化发放率达到100%；全市发放城市“低保”资金1816万元，保障了1.2万户、3.3万人的基本生活。贫困地区困难群众、受灾群众和三峡移民生产生活得到妥善安置，农村低保制度初步建立，失地农民生活得到保障。新建、扩建乡镇社会化供水工程12处，新增供水人口10万人。市区居民人均可支配收入、农民人均纯收入分别达到8883元、3690元，增长14.6%和10.1%。

发展环境有了较大改善。“三城联创”活动富有成效，城市形象、市民文明程度明显提升。城市规划编制、管理工作得到加强，开发建设取得新成绩。泰城重点实施了“四大”基础设施工程和“三项治理”工程建设，南湖公园即将建成，天平湖公园一期建成运营。市高新区发展框架有了新的拓展，落地大项目明显增加，承载能力进一步增强。各县市城市和中心镇的规划建设管理也上了一个新台阶。治理整顿土地市场秩序取得显著成效。启动生态市建设，环境质量明显改善。积极开展行政效能监察和民主评议行风活动，依法整顿和规范市场经济秩序，严厉查处损害发展环境的违法违纪行为，维护了投资者与企业的合法权益。安全生产、来信来访、社会治安综合治理得到加强，“平安泰安”建设取得显著成效。

各位代表，过去的一年，全市上下风正、气顺、心齐、劲足，出现了干事创业氛围浓、经济社会发展加快、城乡面貌显著变化、人民群众得到实惠增多的良好局面，不仅全面完成了市十四届人大二次会议确定的各项目标任务，而且GDP、地方财政收入、全社会固定资产投资等主要经济指标提前一年完成“十五”计划。一年来，我们认真落实市委确定的工作思路，尤其是在以下五个方面用心把握，积极探索，大胆实践。

（一）结合实际认真落实科学发展观。在国家实施宏观调控的新形势下，坚持用科学发展观统揽全局，紧紧扭住发展不放松，以人为本、全面协调、统筹发展；牢固树立“调控是机遇、困难也是机遇”的意识，坚持“四个坚定不移”，创新思维，开动脑筋，潜心研究破解面临的土地、资金、基层组织运转、体制机制障碍等突出难题，赢得了主动，保证了一批“大高外新”项目落地建设，保证了基层组织正常运转和农村公益事业健康发展，经济与社会发展的动力和活力明显增强。特别是全社会完成固定资产投资357.2亿元，增长37.5%，是“九五”期间固定资产投资总额的91.7%。

（二）坚持运用系统的观点调整优化经济结构。坚持量的扩张与质的提高并重，不断加大产业结构、企业组织结构和所有制结构调整力度，尤其是坚持改制、改造、改组并举，大力培植工业主导产业、骨干企业和利税大户，大力发展高新技术产业及其产品，规模企业明显增加，骨干企业综合竞争能力明显增强。全市规模工业企业新增126家；销售收入过亿元、利税过千万元的企业分别达到129家和155家，增加39家和60家，新矿集团销售收入突破百亿元大关；实缴税金过千万元的企业达到61家，增加22家。

（三）集中力量打好招商引资攻坚战。把招商引资作为推进经济实现跨越式发展的关键措施来抓，紧紧抓住国内外产业加速转移的机遇，创新机制，突出重点，分区作战，强力推进，招商引资取得重大突破。全市累计合同利用外资4.5亿美元、实际利用外资3.1亿美元，增长81.4%和103.4%；签订引进国内资金项目1260个，合同引资额388.5亿元、市外到位资金183.7亿元，增长30.2%和53.1%，为加快发展提供了强有力的支持。

（四）加大体制机制创新力度。坚持企业改制与招商重组相结合，多渠道筹措改制成本，尽最大努力实现职工充分就业，最大限度保障职工权益，企业改制得到深化完善。事业单位改革开始启动，市直首批29家经营开发服务类事业单位的改企转制试点稳步推进。成立了市国有资产监督管理委员会，理顺并完善了城市规划、管理、执法体制及土地储备制度，对泰山景区管理体制进行了重大调整。同时，认真抓了统计制度、土地管理、公安管理体制等方面的改革，为发展和稳定奠定了良好的体制机制基础。

（五）不断加强和改进政府工作。以落实《行政许可法》为契机，坚持立党为公，执政为民，恪尽职守，勤奋工作。重视加强“三个体系”建设，深入开展政府提速工作，讲责任、讲质量、讲效率，用心思考、用心工作、用心落实，推动了工作作风的转变和服务质量的提高。积极转变行政管理方式，谋势造势，优化发展环境，搞好公共服务。坚持依法行政，廉洁从政，尽心竭力为群众办实事、解难题，赢得了群众的支持和拥护。

各位代表！过去的一年，全市经济与社会发展取得显著成绩。这是全市各级认真贯彻中央和省决策部署、坚持科学发展观的结果，是市委正确领导，市人大、市政协及社会各界有效监督和大力支持的结果，是全市人民艰苦奋斗、团结拼搏的结果，也是驻泰各单位自觉融入泰安经济和社会发展，尽心竭力，拼搏进取，不断开拓新局面的结果。在此，我代表市人民政府，向全市广大干部群众、各民主党派、工商联、无党派爱国人士和社会团体，向驻泰人民解放军、武警官兵、政法干警和省以上驻泰单位，向关心、支持泰安现代化建设的港澳台同胞、海外侨胞及在我市投资创业的境内外朋友，表示崇高的敬意和衷心的感谢！

各位代表，过去的一年，我们虽然取得一些成绩，但用辩证的观点来审视我们的工作，还存在许多矛盾、困难和问题。经济综合实力还不够强，经济结构性矛盾仍然比较突出，尤其是传统产业所占的比重大，高新技术产业和高附加值制造业所占的比重低，“大高外”项目、骨干企业少，对经济增长支撑带动能力还不够强。以旅游为龙头的服务业发展速度不快、水平不高，比较优势还没有真正凸显出来；农民增收的基础还不牢固，农产品商品率低，农业抗御自然灾害和市场风险的能力不强，农村劳动力转移不快，解决“三农”问题的任务还非常繁重；人才匮乏，尤其是缺乏高层次的技术人才、经营管理人才和各类专业人才，人才的培养、引进、选拔使用、市场配置等有效机制的建立和完善尚需做出更大的努力；制约经济发展的因素还比较多，建设用地、信贷资金等供应仍然偏紧，就业再就业和人口形势依然严峻，部分低收入群众的生活还比较困难；发展环境亟待进一步优化，政府工作人员的思想观念、工作作风、服务质量还有诸多不适应的地方，对坚持科学发展观的认识有待进一步强化，驾驭市场经济的能力还有待于进一步提高，廉政建设、社会治安还存在一些薄弱环节，尤其是一些社会矛盾集中凸显，社会稳定的压力大、构建和谐社会的任务非常繁重等。对此，我们必须有一个清醒的认识，正视矛盾和问题，潜心研究解决的办法和措施，强力推进全市经济和社会更快更好地发展。

二、明确目标任务，努力推进经济与社会全面快速协调发展

根据市委确定的工作思路，今年政府工作的总体要求是：坚持以建设经济强市为目标，继续突出工业经济、民营经济、招商引资三大重点，坚定不移地加快发展、坚定不移地优化结构、坚定不移地抓大项目、坚定不移地保护生态和节约资源；在工作推进方式上，继续坚持党政强力推动与加快市场化相结合，确保经济发展高速度、高质量、高效益、强后劲；全面繁荣发展社会事业，大力加强社会主义精神文明和民主法制建设，促进经济社会全面快速协调发展。主要预期目标是：生产总值增长13%；地方财政收入同口径增长15%；固定资产投资增长30%；实际利用外资增长35%，外贸出口增长20%；新增城镇就业再就业7万人，城镇登记失业率控制在4%以内；人口自然增长率控制在7.4‰以内；居民消费价格总体水平涨幅控制在2%左右；市区居民人均可支配收入增长9%，农民人均纯收入增长6%。实际工作中，力争完成得更好一些、发展得更快一些。

做好今年的政府工作，我们既面临挑战和困难，更有机遇和希望。国内外资本重组、产业转移进程加快，外资准入领域不断扩大，国家宏观调控的积极效果进一步显现；经过多年的改革与发展，市场主体不断培育壮大，经济的内生性自主增长机制不断强化，承接产业转移的能力不断增强，尤其是各级干部特别是领导同志在落实国家宏观调控政策的过程中经受了锻炼，驾驭市场经济和复杂局面的能力进一步提高，全市上下干事创业谋发展、团结拼搏建强市的氛围日益浓厚，广大干部群众越干越想干、越干越敢干、越干越会干。只要我们进一步强化机遇意识、危机意识和创新意识，增强加快发展的紧迫感和责任感，下定决心，鼓足干劲，顽强拼搏，攻坚克难，就一定能够夺取改革与发展的更大胜利。在工作的指导和把握上，要做到“五个坚持”：坚持以科学发展观统揽全局，紧紧抓住执政兴国第一要务不动摇，更好地利用战略机遇期，见机有为，有大作为；坚持以结构调整为主线，加快推进经济增长方式转变，发展循环经济，建设资源节约型社会，努力提高经济运行质量和综合竞争能力；坚持不断推进市场化取向改革，更加注重发挥市场配置资源的基础性作用，加大体制机制创新力度，着力解决影响发展全局的深层次矛盾和问题，使经济社会发展更加充满生机活力和更加富有效率；坚持统筹国内发展和对外开放，抓住我国加入世贸组织进入“后过渡期”、进一步扩大外资准入领域的有利机遇，在更大范围、更广领域、更高层次上参与经济合作与竞争，打造加快发展的新优势；坚持以人为本、构建和谐社会，努力维护好和实现好人民群众的根本利益，保护好、引导好、发挥好广大干部群众的积极性和创造性，形成干事创业、加快发展的强大合力。

三、加大调整优化经济结构力度，努力提高经济运行质量和竞争能力

（一）始终把解决好“三农”问题作为工作的重中之重。严格保护耕地、调整优化结构、推进城乡统筹，促进农业增效、农民增收、财政增长，确保农村稳定。重点抓好“一个深化、两个突破、四项建设”。“一个深化”，就是深化农村改革。稳定完善农村土地承包，引导规范农村土地流转。认真落实农村税费改革、粮食直补和良种补贴政策，加大对农业的支持力度，农业税税率再降低2个百分点。严格农民负担工作考核和一票否决制度，坚决避免农民负担反弹。“两个突破”，一是推进农业结构调整和产业化经营实现新突破。围绕“三个三分之一”的中期调整目标，集中力量培植壮大无公害、绿色、有机农产品，全年新增认证面积分别达到20万亩、10万亩和3万亩。强力推进实施“奶业富民工程”，年末全市奶牛存栏达到10万头。着力培育壮大一批农业龙头企业，加快发展各类农村合作经济组织，加大农业科技创新和推广应用力度，健全完善农业标准、农产品质量安全监测、动植物疫病防治体系。二是推进农村劳务输出实现新突破。进一步加强“三大体系”建设，各级财政要列出专项经费，搞好“订单式”、“定向型”技能培训，提高农民的就业能力和层次，努力打造泰安特色劳务品牌，年内全市新增市外劳务输出8万人。“四项建设”，一是大力加强以治水为重点的农业基础设施建设，重点搞好节水工程、水土保持和大中型水库除险加固；搞好乡镇社会化供水建设，加快“村村通自来水”进程；启动东平湖库区排涝渠系规划建设，加快实施山区、平原、绿色通道和村镇四大绿化工程。二是继续抓好“村村通油路”、“村村通客车”工程建设，年内确保基本实现“两通”目标。三是大力抓好农村医疗卫生保障体系建设，市财政投入1000万元扶持资金，全面实行农村新型合作医疗。四是加强乡村财源建设，强化措施，拓宽视野，多管齐下，坚持增收、节支、减人、减事和转移支付并举，确保基层组织正常运转和以教育、卫生为重点的农村公益事业加快发展。同时，进一步加强农业综合开发，大力发展农业机械化，努力提高农业综合生产能力。

（二）加快培植壮大工业经济。坚持工业立市不动摇，以改革为动力，以结构调整为主线，按照“培植一批支柱产业、一批大型企业、一批知名品牌”的要求，坚持“三改两上”，即实施改革、改组、改造，促进工业经济上规模、上水平。一是深化企业改革。按照既定的思路和要求，未改制的企业和虽已改制，但不到位、不完善的企业，年内都要完成改制和深化改制的工作。引导已改制企业，按照建立现代企业制度的要求，创新内部运行机制，进一步激发企业加快发展的内在动力和活力。二是加大企业改组力度。进一步解放思想，加大招商引资、合资合作力度，对企业实施整体、分块重组，做大做好借助外力、激活内力的文章。依托现有大企业和经济园区，搞好资本、技术、人才等要素、资源的整合，注重培育高端产品和特色产业，依托优势产品拉长产业链。继续坚持“政府推进，市场运作，借助外力，高点起步”，加大六大主导产业培植力度，力争汽车、电工电器、无机非金属新材料和精细化工四个产业率先实现大的突破。三是大力推进技术改造。瞄准世界先进水平，积极运用高新技术和先进适用技术对传统产业进行技术改造，不断提升企业的技术装备水平、管理水平和市场竞争能力，重点抓好76个投资过亿元的技改项目建设。四是加快推进工业经济上规模、上水平。加快推进技术创新，加强以企业家为主体的企业人才队伍建设，调整优化经济结构，实施名牌战略，促进工业经济总量膨胀和质量效益提升，着力培植一批主业突出、竞争力强、对财政支撑和对地方经济拉动作用明显的大企业、大集团，重点抓好30强企业的发展壮大，增强企业核心竞争力。确保利税过千万元的企业达到175家，销售收入过10亿元的达到10家，过50亿元的达到2家；高新技术产业、产品产值占规模以上工业总产值的比重均提高2个百分点以上。

（三）强力推进旅游服务业快速发展。坚持把以旅游业为龙头的服务业作为加快发展的主攻方向，加快思想观念、体制机制和经营理念创新，推动旅游服务业实现大突破。一是加大旅游服务业体制创新力度。在搞好试点的基础上，加大推进力度，全面推开市、县市区经营开发服务类事业单位改企转制工作，激发旅游服务业快速发展的内在动力。二是加快培植旅游支柱产业步伐。继续坚持“四大”思路，以满足游客需求、延长游客滞留时间为目标，高起点修编完善旅游发展规划，突出特色，打造精品，搞好旅游资源、产品的整合开发，逐步形成以泰山为中心、辐射周边、具有规模效应和竞争力的大旅游格局。重点抓好泰山博物馆、汉御道文化、泰山国际休闲运动中心、宝龙城市广场和东平湖水浒文化旅游区、徂徕山综合开发、莲花山等景区和项目的开发建设，规划搞好旅游各要素配套设施建设和旅游商品、特色小吃开发，精心策划推出一批商务会所、康体休闲、宗教文化等特色旅游。鼓励民间资本和外资参与旅游企业改组改造，积极扶持创办国际旅行社和成立合资、外商独资的旅行社，鼓励有实力的旅行社到外地设立分支机构、加入各种形式的旅游联合体，增强招徕能力。进一步细分市场，强化宣传促销，努力拓宽国内外客源市场。强化综合执法，加大专项整治与综合整治力度，严厉打击违法经营、欺客宰客、强拉强卖等扰乱市场秩序、有损泰山形象的行为。三是加快发展服务业。大力发展商贸流通、现代物流业，加快培育壮大一批规模大、集聚力强的名牌骨干企业，重点抓好银座商城扩建、光彩大市场续建、品牌港建设和泰山国际保税物流中心及各类配送中心建设。繁荣提升餐饮娱乐业，突出特色、中外结合，整合提升现有宾馆、酒店的档次，改造和规划建设4-5处四、五星级宾馆，扶持发展几处西餐馆和日韩料理；以弘扬泰山文化、民族文化和现代文化为主题，搞好休闲文化娱乐一条街和演出广场规划建设，力争年内泰城有较高档次和品位、系统性、经常性的演出，实现旅游产业与文化产业良性互动。加快发展社区服务业，大力培育发展以家政、维修、医疗、健身、科技、教育、就业中介为主要内容的社区服务，力争年内有实质性进展。积极发展交通服务业，重点发展客、货运输，搞好老汽车站改扩建和停车场规划建设，积极发展汽车维修、养护、二手车交易和租赁等服务业。大力发展中介服务业，制定落实鼓励政策，扶持现有中介机构做大做强，鼓励中介机构实行不受地域和所有制限制的联合、重组，培育形成一批实力强、信誉好、有特色的科技、咨询、会计、法律等中介服务机构。大力发展土地、房产、建筑、劳动力、技术等要素市场，促进各类生产要素的合理流动和优化配置。重视发展农村服务业，整合现有农业服务机构组织，开展连锁经营，实行“超市下乡”，为农民搞好生产、技术、加工、销售、消费品供应和劳务输出等服务。进一步放宽市场准入，认真清除行业垄断、收费标准不合理等障碍，鼓励和放手让民营资本、外来资本等多种所有制经济成分在更广泛的领域参与服务业的竞争与发展，促进全市服务业上档次、上水平。

（四）加快推进城镇化和城乡一体化进程。围绕建设“蓝天、碧水、青山”绿色家园的目标，坚持城乡统筹和“四高”要求，加大规划建设力度，提高经营管理水平，努力打造独具特色、富有生机和魅力的现代化山水园林旅游城市。一是进一步强化规划的刚性作用。完善规划和建筑设计招投标、专家论证、公众参与、重大规划公示制度和规划决策审批程序，强化精品意识，高起点、高标准、高质量地抓好新一轮城市总体规划修编和各项专业规划、景观改造规划、详细规划的编制，同时把乡村规划纳入整体规划范围，努力提高规划辐射率和覆盖率；加强规划的集中管理、控制和实施监督，坚决制止零星低档次房地产开发，刹住乱搭乱建的歪风，利用规划整合资源、保护资源，实现资源的良性循环。二是加大城镇开发建设力度。按照“严格保护泰山景区，改造提升老城区，开发建设新城区”的思路，突出生态市建设和环境改善，加快搞好泮河大街建设、时代发展线延伸、泮河综合整治等重点续建工程，集中力量搞好老泰莱路城区段改造、泰山东苑基础设施、开元路等新开工项目建设。搞好房屋普查，抓好经济适用房、廉租房建设。统一规划、市场运作，加快推进城中村改造步伐。搞好县市城市和中心镇、小城镇开发建设，努力提高城镇化水平。突出项目引进、基础设施完善和体制机制创新，加快市高新区、旅游经济开发区和县市区经济开发区、高新区建设，提高运行质量，增强载体功能。三是切实加强城市管理。坚持以人为本、标本兼治、点面结合、市区联动，大力实施城市环境综合整治，重点搞好东岳大街、泰山大街等六条主干道和相关路段、重点部位节点的整治，加大对小街巷、旧小区的改造和整治力度，加强社区建设和管理，努力创造一个“绿亮净美”、整洁有序的城市环境。完善创新城市管理长效机制，切实提高管理水平。培育发展物业管理企业，积极探索市场化加强物业管理的有效途径和办法。四是进一步做好经营城市的文章。创新体制，搭建起政府经营城市的平台，搞好资源整合与运营；收缩政府直接投资，扩张市场融资，完善实施项目法人责任制、“代建制”等，大力推进城市建设投资主体多元化、市场化、社会化；进一步完善经营性用地招标、拍卖、挂牌出让制度，搞好存量、增量土地的储备与有序出让，规范土地市场秩序，提高政府土地收益。

（五）充分发挥投资和消费对经济增长的拉动作用。努力做好扩大招商引资、争取金融机构扩大信贷投放、企业自身积累、启动民间资本、国债项目对接争取、资本市场融资等文章，千方百计增加建设投入。重点抓好3－5个企业在境内外主板和创业板上市，争取实现企业境外上市零的突破。坚持集中资金保重点、办大事，优化资金投向，加大对经济增长支撑作用明显、拉动能力强的大项目建设投资力度，确保泰山抽水蓄能电站一期第一台机组建成投产，集中力量抓好石横电厂扩建、济菏高速公路东平段等300多个投资过亿元的大项目建设。同时，根据国家产业政策，发挥优势，突出重点，依托高层次专家咨询机构，有针对性地策划论证一批投资数额大、回报率高、短期内能开工建设的大项目、好项目，力争年内策划论证投资过亿元的项目突破100个。努力促进消费增长，通过加快经济发展、促进广泛就业、完善收入分配调节机制、健全社会保障制度等措施，不断增加城乡居民特别是中低收入群众和特困居民的收入，提高居民的消费预期和消费能力；优化消费环境，引导人们转变消费观念，更新消费方式，拓展消费领域，扩大消费信贷，加快培育和扩大住房、汽车、电子信息等消费热点，推动消费结构优化升级和消费需求持续增长。

（六）加强和改进财税金融工作。坚持生财有道、聚财有方、科学理财，深化财税体制改革，大力培植地方财源，努力提高财政收入占GDP、税收收入占财政收入“两个比重”。一是拓宽财政增收渠道。通过调整经济结构、培植利税大户、搞活国有资产经营、做好经营城市和以地生财的文章等途径，夯实财政增收的基础。依法加强税收征管，强化税源监控，实行社会综合治税，切实把经济发展的成果反映到财政增收上来。二是优化财政支出结构。深化“收支两条线”改革，进一步完善部门预算和政府采购制度，推进国库集中支付，强化财政监督，努力提高财政支出效益。在严格保工资、保稳定、保法定支出的基础上，重视加大对“三农”、社会保障、教育卫生等领域的投入，加大转移支付力度，大力压缩非生产性开支，确保实现财政收支平衡。三是充分发挥金融对经济发展的促进作用。积极支持国有商业银行和其他各类金融机构的改革与发展。继续深入开展“创建金融安全区”活动，搞好企业信用评级，加快担保体系建设，加大银地、银企合作力度，提高合作的规模和档次。抓住国有商业银行改革的时机，运用好金融资产管理公司的各项政策，加快不良资产的处置。积极发展保险、证券业务，加快城市信用社申办商业银行步伐，办好地方性金融机构，更好地服务于经济发展。

四、加大改革开放力度，进一步激发经济发展的动力与活力

(一)继续深化改革。一是深化国有资产管理体制改革。按照国有资产监督管理委员会组建方案要求，加快职能整合，严格依法履行出资人职责，实行管资产与管人、管事相结合，健全完善国有资产监督管理体系。进一步创新国有资产运营机制，积极探索国有资产经营的新路子，严格经营业绩考核，实现国有资产保值增值，推进国有经济布局和结构战略性调整。二是深化投融资体制改革。按照“谁投资、谁决策、谁受益、谁承担风险”的原则，以放活企业投资自主权为重点，逐步建立起市场引导、企业自主决策、银行独立审贷、融资方式多样、中介服务规范、宏观调控有效的新型投资体制。建立健全政府投资项目决策机制和投资评价、责任追究制度，切实提高投资效益。三是深化粮食流通体制和国有粮食企业改革。完善市场准入制度，加强粮食市场监督和管理，鼓励多种经济主体进入粮食市场。强化地方粮食储备，完善市场风险预警机制和粮食安全、价格应急预案，增强政府宏观调控和应对突发事件的能力。多渠道筹措改制成本，妥善分流安置富余人员，加快实施国有粮食企业改制步伐，增强发展活力。

(二)大力推进非公有制经济加快发展。认真落实各项扶持政策，着力创造平等竞争、一视同仁的法制、政策和市场等环境，推动民营经济扩规模、上档次。进一步支持引导民营企业进入更多的领域和行业，参与国有、集体企业的改制重组，推动非公有制企业体制、机制、管理和技术创新，扶持发展一批企业家素质高、核心竞争力强的名牌企业。坚持政府引导、市场运作，加快建立融资担保、信用评价、人才培训、技术信息、法律服务和行业协会等服务体系，切实解决民营经济发展中遇到的融资、技术、信息等困难和问题。鼓励各类人才创办、领办和从事咨询服务业，为民营企业提供创业指导、项目评估、投融资和技术咨询等服务。继续加大对规模企业和纳税大户的培植力度，重点抓好100个投资过亿元的民营工业项目建设,年末民营经济实现增加值和实缴税金均增长30%以上。

(三)进一步提升对外开放水平。坚持“市外就是外”，充分发挥比较优势，更加积极主动地承接国内外资本产业转移，努力提高利用外资的规模、质量和水平。继续以珠江三角洲、长江三角洲和日韩、美国、港澳台、欧洲、东南亚“五大区域”为重点，分工负责，整体推进，重点突破，全面加强与国内外大企业特别是世界500强的合资合作，积极吸引跨国公司在我市设立研发、加工和采购中心，做大做足借助外力、加快发展的文章。进一步创新招商机制和方式，继续坚持“六个结合”，充分发挥“五个作用”，整合力量，形成招商引资的整体规模效应。高度重视抓好招商引资项目的跟踪服务和落实工作，确保项目早落地、早见效。高度重视抓好环境建设，为现有外资企业提供优良的服务，使其扩大规模，做好以商招商的工作。继续实施以质取胜和市场多元化战略，大力开拓市场，加快调整出口商品结构，突出抓好机电、成套设备及农副产品出口和高新技术、高附加值产品出口。大力开展境外投资合作、承包工程和劳务合作等业务，努力提高经济外向度。

五、坚持以人为本，努力构建社会主义和谐社会

大力发展科技、教育等项社会事业。深入实施“科教兴泰”和“人才强市”战略。以发展高新技术产业为主线，创新管理体制，多元增加投入，加快构建以高校和科研单位为依托的应用科学研究体系、以企业为主体的技术创新体系、以中介机构为主体的科技服务体系；加强与国内外高校、科研机构的科技合作，建立完善产学研协调发展机制，加强知识产权保护；大力推广应用新技术、新成果，努力提高经济与社会发展的科技含量。进一步完善以县为主的农村义务教育管理体制，增加投入，加大教育教学改革、农村中小学危房改造、规范化学校和教育信息化建设力度，积极开展教育示范县和教育强乡镇创建活动，大力推进“双高普九”，积极探索社会化办学的有效途径，加快发展高中教育、学前教育、职业教育和成人教育。大力支持驻泰高校的发展与改革，着力抓好泰山学院二期工程、泰山职业技术学院配套完善。进一步强化“人才是第一资源”的意识，创新体制，转变观念，紧紧抓住培养、吸引、使用三个环节，建立完善鼓励优秀人才脱颖而出、留住人才、充分施展才干的有效机制和良好环境，尤其是重视各类高层次人才的培养和高级技工队伍建设，为加快发展提供人才保障。大力开展爱国卫生运动，深化城乡医疗卫生、卫生监督和药品生产流通等体制改革，加快中医药现代化发展步伐，全面完成市、县两级疾控中心和传染病医院建设，提高应对突发公共卫生事件的能力。加快发展文化产业，净化文化市场，加强文物保护，繁荣发展文艺创作、艺术表演和新闻出版、广播电视事业，年内开工建设档次高、功能全的广播电视大楼。加强体育设施建设，提升全民健身和竞技体育水平。认真贯彻《宗教事务条例》，依法加强对宗教事务的管理，认真落实民族政策，加快发展少数民族经济，推进民族团结与进步。进一步搞好国防教育、人民防空和民兵预备役建设，重视做好优抚、安置工作，推动双拥共建活动深入开展。搞好统计、普查工作，关心支持妇女儿童、残疾人、老龄等工作，加快发展社会各项事业。

推进经济与人口、资源、环境协调可持续发展。积极应对第四次人口出生高峰，着力建立完善依法管理、村（居）民自治、优质服务、政策推动、综合治理工作新机制，坚持严格责任、严格考核、严格纪律，对工作不力、突破年度人口控制计划的，坚决实行重点管理、一票否决，努力稳定人口低生育水平。大力发展循环经济，加快建设资源节约型社会，坚持资源开发与保护节约并重，严格保护耕地、水、矿产资源，加强河沙资源管理，全面开展节能降耗和资源综合利用；以煤炭、电力、建材等资源依赖型行业为重点，引导企业积极采用先进技术和行业先进标准，努力提高资源利用效率；加快建立工业固体废弃物、城市生活垃圾和废旧物资的回收、加工、利用体系，提高资源循环利用水平。突出植树造林和治污两大重点，大力推进生态市建设，尤其是严格执行对新建项目的环境影响评价和“三同时”制度，强化污染源头控制；全面实施排污许可制度，严格控制污染物排放总量和实现达标排放，突出搞好重点流域和区域污染治理，特别是乘省实施“两湖一河”碧水行动计划的东风，加大对东平湖、大汶河的综合治理力度，推进城乡环境保护和生态建设良性循环，努力争创“国家环保模范城市”。

切实维护好广大人民群众的切身利益。“德莫高于爱民，政莫重于利民”。坚持“群众利益无小事”，倾情尽力解决好事关群众切身利益的实际问题。继续坚持“市场调节就业、劳动者自主择业、政府促进就业”的方针，重视发展劳动密集型产业，千方百计增加就业岗位。认真落实小额担保贷款、免费培训、岗位补贴、税费减免等优惠政策，加大就业特困人员再就业帮扶力度，努力降低失业率，提高就业率，实现最大限度就业。加大财政支持力度，加快完善以养老、失业、医疗等保险为主要内容的社会保障制度，积极推进做实养老保险“个人账户”、“企业年金”和失地农民养老保险试点工作，努力提高保障能力。高度关注抓好对困难、弱势群体的社会救济和受灾群众的生活救济，抓好农村养老保险、慈善捐助及专项社会救助等，逐步构筑起功能比较完善的社会救助体系。适当提高城乡低保标准，做到应保尽保。加快建立财政供养、“四级”联保的五保户供养新机制。重视处理好土地征用、房屋拆迁、企业改制、职工和农民工工资拖欠等涉及群众切身利益的焦点、难点等问题。

加强精神文明和民主法制建设。加强社会主义思想道德建设，大力弘扬以爱国主义为核心的民族精神和以改革创新为核心的时代精神。深入开展“三城联创”等群众性精神文明创建活动，大力倡导遵纪守法、明礼诚信、团结友善、敬业奉献、见义勇为等良好的社会风尚。认真执行人大及其常委会的决议、决定，自觉接受人大的法律监督和政协的民主监督，主动听取各民主党派、工商联、人民团体和社会各界人士的意见和建议，认真做好人大代表建议和政协委员提案办理工作。重视加强基层民主政治建设，积极推进政务公开、厂务公开和村务公开，拓宽反映社情民意的渠道。继续深入开展普法宣传教育和依法治理工作，搞好“四五”普法总结验收，努力争创全国普法工作先进市。深入开展“平安泰安”建设，坚持打防并举，完善落实责任制，大力加强社会治安综合治理，重点打击各种严重暴力犯罪、流氓恶势力犯罪、各种形式的恐怖犯罪和盗窃、抢劫等多发性侵财犯罪活动，进一步增强人民群众的安全感。重视做好人民来信来访工作，及时处理群众反映的突出问题，化解矛盾，维护稳定。强化安全生产基层、基础工作和目标管理责任制的落实，严防各类重特大事故的发生，确保国家和群众生命财产安全。

六、加快推进政府转型，进一步优化发展环境

新形势、新任务，对政府工作提出了新的更高的要求。各级政府必须按照为民、务实、清廉、创新的要求，坚持立党为公、执政为民，加快建设责任政府、阳光政府、服务政府、法治政府；充分认识环境建设的极端重要性、长期性和艰巨性，加大力度，常抓不懈，努力营造加快发展的良好环境。

(一)努力提高执政服务水平。建立市级政府决策和重点项目、旅游、城市规划等专家咨询委员会，完善社会公众参与、专家咨询论证和政府决策相结合的决策机制。认真贯彻落实《全面推进依法行政实施纲要》，自觉学法、懂法、用法，健全完善执法责任制、错案责任追究制和行政效能监察、举报投诉处理等一系列规章制度，做到有权必有责、用权受监督、侵权须赔偿、违法要追究。积极推进综合执法，加强执法队伍建设，强化执法监督检查，切实提高行政执法水平。重视加强对公务员各方面理论、知识的学习培训，不断掌握新知识，积累新经验，增长新本领，全面提高公务员队伍素质。

(二)加快政府职能转变。积极推进政府管理创新，加快建设融行政审批、政府采购、效能投诉、市长公开电话于一体的政务服务中心，以电子化、网络化、信息化带动政府工作的规范化，最大限度地集约利用行政资源，加大对市高新区的授权，支持高新区体制创新，千方百计提高办事效率和服务质量，使服务对象进一个门，办妥一切需要办理的事情。深化行政管理体制改革，严格控制机构、编制，真正把政府职能转到经济调节、市场监管、社会管理和公共服务上来，做到有所为、有所不为，不越位、不错位、不缺位。坚持行政强力推动与加快市场化相结合，转变经济管理方式，强化为市场主体服务和创造良好环境的责任，提高按市场经济规律管理经济、充分发挥市场主体作用和主要运用经济、法律手段调节经济运行的意识和能力。更加注重履行社会管理和公共服务职能，加快建立健全各种突发事件的应急机制，提高应对公共危机的能力。

(三)树立清正廉洁形象。认真执行领导干部廉洁自律的各项规定和党风廉政建设责任制，严厉查处违纪违法案件，坚决纠正部门和行业不正之风。积极构建与社会主义市场经济体制相适应的教育、制度、监督并重的惩治和预防腐败体系，通过加强思想道德、纪律和法制教育，牢记使命，常想为政之本，常修为政之德，常思贪欲之害，常怀律己之心，常省自身之过，筑牢拒腐防变的思想防线；加强廉政制度建设和监督制约，规范约束行政行为，形成权力正确行使的有效机制。坚持抓源治本，加强监督体制建设，从源头上预防和治理腐败。始终保持谦虚谨慎、不骄不躁和艰苦奋斗的作风，牢固树立正确的世界观、人生观、价值观、政绩观，履行好廉政承诺，廉洁从政、从严治政，以廉洁勤政的良好形象，赢得人民群众的信赖和支持。

(四)建立完善高效落实机制。崇尚实干、不尚空谈，坚决反对和防止官僚主义、形式主义。紧紧围绕责任、质量和效率，进一步健全完善决策目标、执行责任、考核监督三个体系和快捷高效的落实机制，加大对工作特别是重点工程、重大事项、重要工作的责任落实、调度督查和考核奖惩力度，确保各项工作落到实处，抓出大的成效。进一步转变作风，大兴调查研究之风，用系统的、辩证的、开放的观念和改革的思路、市场的办法，研究解决发展中的矛盾和问题。坚持以科学发展观为指导和科学化、民主化、法制化的原则，谋划编制好国民经济和社会发展“十一五”计划，描绘好今后五年的发展蓝图。全体政府工作人员要振奋精神，以强烈的事业责任感、昂扬向上的精神状态和埋头苦干的作风，努力推进全市经济实现跨越式发展和社会全面进步，让群众得到更多的实惠。

各位代表！泰安正处在实现跨越式发展的关键时期，时代赋予我们重任，人民寄予我们厚望。让我们紧密团结在以胡锦涛同志为总书记的党中央周围，高举邓小平理论伟大旗帜，坚持以“三个代表”重要思想为指导，在市委的坚强领导下，坚定信心，抢抓机遇，干事创业，奋力拼搏，在新的一年里创造更加辉煌的业绩，为加快建设经济强市进程、全面建设小康社会而努力奋斗!
